# TFEB Modulates p21/WAF1/CIP1 during the DNA Damage Response

**DOI:** 10.3390/cells9051186

**Published:** 2020-05-10

**Authors:** Sandra Pisonero-Vaquero, Chiara Soldati, Marcella Cesana, Andrea Ballabio, Diego Luis Medina

**Affiliations:** 1 Telethon Institute of Genetics and Medicine (TIGEM), High Content Screening facility, Via Campi Flegrei 34, 80078 Pozzuoli, Italy; spisv@unileon.es (S.P.-V.); c.soldati@tigem.it (C.S.); m.cesana@tigem.it (M.C.); ballabio@tigem.it (A.B.); 2 Deparment of Advance Biomedical Science, Federico II University, 80138 Naples, Italy; 3 Medical Genetics Unit, Department of Medical and Translational Science, Federico II University, 80138 Naples, Italy; 4 Baylor College of Medicine, Houston, TX 77030, USA; 5 Jan and Dan Duncan Neurological Research Institute, Texas Children’s Hospital, Houston, TX 77030, USA

**Keywords:** TFEB, p21, DNA-damage response, cell cycle, autophagy, cancer, genotoxic stress, doxorubicin

## Abstract

The MiT/TFE family of transcription factors (MITF, TFE3, and TFEB), which control transcriptional programs for autophagy and lysosome biogenesis have emerged as regulators of energy metabolism in cancer. Thus, their activation increases lysosomal catabolic function to sustain cancer cell growth and survival in stress conditions. Here, we found that TFEB depletion dramatically reduces basal expression levels of the cyclin-dependent kinase (CDK) inhibitor p21/WAF1 in various cell types. Conversely, TFEB overexpression increases p21 in a p53-dependent manner. Furthermore, induction of DNA damage using doxorubicin induces TFEB-mediated activation of p21, delays G2/M phase arrest, and promotes cell survival. Pharmacological inhibition of p21, instead, abrogates TFEB-mediated protection during the DNA damage response. Together, our findings uncover a novel and direct role of TFEB in the regulation of p21 expression in both steady-state conditions and during the induction of DNA-damage response (DDR). Our observations might open novel therapeutic strategies to promote cancer cell death by targeting the TFEB-p21 pathway in the presence of genotoxic agents.

## 1. Introduction

p21/WAF1/CIP1, hereafter p21, has been originally identified as an important effector of the tumor suppressor gene p53. p21 is a universal inhibitor of the cyclin kinases (Cdks) that are necessary for cell cycle progression. However, accumulating evidence also indicates that it can play a role in a variety of cellular functions such as the regulation of transcription, DNA repair, and the modulation of apoptosis [1,2,3,4]. p21 protects cells against apoptosis elicited by various stimuli, presumably to increase cell viability during the reparation of damaged DNA in growth-arrested cells. As the p21 protein has been shown overexpressed in many tumor types, its anti-apoptotic activity may attenuate the efficacy of therapeutic regimens relying on the induction of apoptosis. This pro-oncogenic activity of p21 has been demonstrated for prostate and breast cancer, gliomas, cervical and ovarian carcinomas, and some other tumors, where its increased expression is frequently associated with unfavorable prognosis and poor survival [5,6]. Thus, p21 has been involved in both tumor-suppressive and oncogenic properties [3,7,8,9]. In pigmented cells, the transcription of p21 is activated by MITF, a master regulator of melanocyte differentiation and proliferation [1]. Paradoxically, overexpression of MiT members, including MITF TFEB and TFE3, and upregulation of their transcriptional gene networks can drive tumorigenesis in different tissues [10,11,12]. Thus, chromosomal translocations involving TFEB and TFE3 have been found in patients with clear renal cell carcinoma (RCC) [13,14,15,16,17,18,19]. In last years, the MITF subfamily of bHLH-zipper transcription factors has been also involved in the positive regulation of a gene-network encoding for proteins implicated in lysosomal biogenesis and autophagy [20,21,22]. Interestingly, the MiT/TFE-dependent autophagic process is enhanced and essential for renal cell carcinoma, melanoma, and pancreatic ductal adenocarcinoma growth by rising the intracellular amino acid pools through the increase in lysosomal catabolic function [23,24,25]. In addition, emerging evidence suggests that MITF transcription factors may play a more direct role in the regulation of the cell cycle. Thus, TFE3 and TFEB contribute to sustaining p53-dependent response by stabilizing p53 protein levels [26]. Furthermore, TFEB depletion halts proliferation at the G1-S transition by inhibiting the CDK4/Rb pathway [27].

While studying the effects of TFEB modulation in cell cycle proteins, we found that the depletion of TFEB severely impairs the basal expression levels of p21, while its overexpression transcriptionally upregulates p21 through direct binding to its promoter in a p53-dependent manner. Most interestingly, pharmacological induction of DNA damage using doxorubicin can induce TFEB/p21 pathway, uncovering a physiological role of TFEB in regulating the cell cycle during stress responses. Thus, TFEB activation can promote G1 arrest through p21 upregulation, delaying G2/M phase arrest, and increasing cell survival upon chemotherapeutic treatment of cancer cells. These observations envisage novel therapeutic strategies in cancer therapy by targeting the TFEB-p21 pathway in combination with genotoxic drugs.

## 2. Materials and Methods

### 2.1. Cell Culture and Treatments

HeLa and ARPE-19 cells were purchased from ATCC. ARPE-19 is a spontaneously immortalized cell line of human retinal pigment epithelium. These cells are diploid and non-transformed. HeLa TFEB and TFEB/TFE3 knockout and p53-null human osteosarcoma (SAOS-2 p53-null) cells were kindly provided by Richard J. Youle (National Institutes of Health, Bethesda, MD, USA) and Dr. Caterina Missero (Centro di Ingegneria Genetica e Biotecnologie Avanzate, Naples, Italy), respectively. HeLa cells stably expressing TFEB-GFP or TFEB 3xflag were obtained as previously described [22,28]. Cells were cultured at 37 °C and 5% CO2 in DMEM (Sigma-Aldrich, St. Louis, MO, USA, D6429) supplemented with fetal bovine serum (10%) and Pen/Strep (1%). Treatments with doxorubicin (D1515, Sigma, St. Louis, MO, USA, D6429), Torin (Tocris, Bristol, UK), or UC2288 (ab146969, Abcam, Cambridge, UK) were performed at indicated time points and concentrations and compared with control DMSO. For colony formation assay, about 200 cells per well were seeded in a 6-well plate. Next day cells were treated, and maintained in culture 8 days more, replacing medium every three days.

### 2.2. Plasmid and siRNA Transfection

Cells were transfected using Trans-IT reagent (Mirus-Bio, Madison, WI, USA) according to the manufacturer’s protocol with plasmids encoding TFEBS211A x3flag [22], CMVp53 (provided by Dr. Caterina Missero), flag p21 (Addgene, Watertown, MA, USA), or 3xflagCMV14 empty (Sigma-Aldrich St. Louis, Missuri, USA). siRNA transfection was performed with Lipofectamine RNA iMAX (Invitrogen, Carlsbad, CA, USA) following the manufacturer’s protocol. Silencing was performed for 72 h at 20 nM with either p21-targeting siRNA (L-003471-00-0005, Dharmacon, Lafayette, Colorado, USA) or non-targeting siRNA (D-001810-10-50, Dharmacon, Lafayette, CO, USA) as the negative control.

### 2.3. RT-qPCR

Total RNA was purified from cells by using the RNeasy Mini Kit (Qiagen, Hilden, Germany). Reverse transcription was performed using the QuantiTect Reverse Transcription Kit (Qiagen, Hilden, Germany). qPCR was performed with a LightCycler® System 2.0 (Roche Applied Science, Penzberg, Germany), using SYBR Green PCR master mix (Applied Biosystems, Foster City, CA, USA) and the following primers: p21 (fw: ggaagaccatgtggacctgt, rev: ggcgtttggagtggtagaaa), p53 (fw: aaggaaatttgcgtgtggag, rev: tgtagttgtagtggatggtgg), TFEB (fw: caaggccaatgacctggac, rev: agctccctggacttttgcag), HPRT (fw: tggcgtcgtgattagtgatg, rev: aacaccctttccaaatcctca). Relative gene expression was calculated by the 2^-ΔΔCt^ method. HPRT was used as the reference gene for the normalization of target gene expression levels. 

### 2.4. Western Blot Analysis

Cells were rinsed twice with cold PBS, resuspended in lysis buffer (10 mM Tris-HCl pH 8, 0.2% SDS) containing protease (Roche, Basilea, Switzerland) and phosphatase (Sigma, St. Louis, MO, USA) inhibitors, and sonicated. Protein concentration was determined by the Bradford method. Then protein samples were separated by SDS–PAGE, transferred to nitrocellulose membranes, and immunoblotted with specific primary antibodies followed by peroxidase-conjugated anti-rabbit or anti-mouse secondary antibodies (Millipore, Burlingtone, MA, USA). Chemiluminescence was detected by a chemiluminescence imager (Amersham Imager 600, Amersham, Little Chalfont, UK). Densitometric quantification of bands was performed using ImageJ (NIH). Primary antibodies used were: TFEB (#4240, Cell Signaling, Denvers, MA, USA), p53 (#9282S, Cell Signaling Denvers, MA, USA), p21 (#2947, Cell Signaling, Denvers, MA, USA), pWee1(Ser642) (#4910, Cell Signaling, Denvers, MA, USA), Myt1 (#4282, Cell Signaling, Denvers, MA, USA), cyclin B1 (#12231, Cell Signaling, Denvers, MA, USA), aurora B (ab2254, Abcam), pHH3(Ser10) (#3377, Cell Signaling, Denvers, MA, USA), cyclin E2 (#4132, Cell Signaling, Denvers, MA, USA), pCDC2(Tyr15) (#4539, Cell Signaling, Denvers, MA, USA), cyclin A (#4656, Cell Signaling, Denvers, MA, USA), p18 (#2896S, Cell Signaling, Denvers, MA, USA), and β-actin (sc-47778, Santa Cruz Biotechnology, Dallas, TX, USA), Caspase-3 (#9662, Cell Signaling, Denvers, MA, USA), pULK757 (#6888, Cell Signaling, Denvers, MA, USA), ULK1 (#8054, Cell Signaling, Denvers, MA, USA), pP70 (#9205, Cell Signaling, Denvers, MA, USA, P70 (#2708, Cell Signaling, Denvers, MA, USA), 4EPB (#9644, Cell Signaling, Denvers, Massachusetts, USA), p4EPB (#9456, Cell Signaling, Denvers, MA, USA), TFE3 (#14779, Cell Signaling, Denvers, MA, USA). 

### 2.5. Chromatin Immunoprecipitation (ChIP) Assay

Chromatin immunoprecipitation assay in HeLa cells transfected with either 3xflagCMV14 empty or TFEB S211A 3xflag plasmids and in HeLa cells stably expressing TFEB 3xflag was performed as previously described [29]. Anti-flag antibody (F7425, Sigma, St. Louis, MO, USA) was used to immunoprecipitate TFEB. qPCR was performed with a LightCycler® System 2.0 (Roche Applied Science, Penzberg, Germany), using SYBR Green PCR master mix (Applied Biosystems, Foster City, CA, USA) and the following primers: p21 promoter set 1 (fw: acaactcactcgtcaaatcc, rev: caatctccctacaccctaca), p21 promoter set 2 (fw: gggcggttgtatatcagg, rev: ctccacaaggaactgactt), ATP6V1H promoter (fw: tcgggaatccagttgtccg, rev: gccgcacaggtagaaggaa), HPRT promoter (fw: gccacaggtagtgcaaggtctt, rev: ttcatggcggccgtaaac). Primers for ATP6V1H and HPRT promoters were used as positive and negative controls, respectively [30].

### 2.6. Cell Cycle Analysis by Flow Cytometry

HeLa cells were harvested and resuspended in PBS. For the fixation, 9-fold volume of ethanol 70% was added and incubated a 4 °C for at least 1 h. Next, cells were centrifuged, washed in PBS and resuspended in PBS containing RNase A 0.1 mg/ml. After incubation for 1 h at 37 °C, propidium iodide was added to a final concentration of 10 µg/ml and samples were analyzed in a flow cytometer Accuri C6 or with BD FACSAriaIII.

### 2.7. High Content Imaging Analysis

Cell cycle analysis by high content imaging was performed by using the Click-iT Plus EdU Alexa Fluor 488 Imaging Kit (Life Technologies, Carlsbad, CA, USA) according to manufacturer instructions. In addition, immunofluorescence detection of histone phosphorylation to identify cells in mitosis was performed by using an anti-pHH3 (Ser28) antibody (H9908, Sigma, St. Louis, MO, USA), in combination with an Alexa Fluor 647 anti-rat antibody (Life Technologies, Carlsbad, CA, USA). Images were acquired with an automated confocal microscopy (Opera System, Perkin Elmer, Walltham, MA, USA) and analyzed through Columbus Image Data Storage and Analysis System (Perkin-Elmer, Walltham, MA, USA), by applying an elaborated script. In essence, cells in S-phase, which have incorporated EdU (5-ethynyl-2’-deoxyuridine, a nucleoside analog of thymidine), are visualized in green; cells with low Hoechst 33342 sum intensity (low DNA) and EdU/pHH3 negative are G1-phase cells; cells with high Hoechst 33342 sum intensity (high DNA) and EdU/pHH3 negative are G2-phase cells; finally, cells in mitosis are visualized in red (pHH3 positive). Low/High Hoechst 33342 sum intensity threshold is calculated by the quantification of Hoechst 33342 sum intensity mean in EdU positive nuclei. Immunofluorescence detection of endogenous TFEB was performed by using the antibody anti-TFEB (#4240, Cell Signaling, Denvers, MA, USA). Alexa Fluor 488 anti-rabbit antibody (Life Technologies, Carlsbad, CA, USA) was used to detect the anti-TFEB antibody. Images were acquired with an automated confocal microscopy (Opera System, Perkin Elmer) and analyzed through Columbus Image Data Storage and Analysis System (Perkin-Elmer, Walltham, MA, USA). A dedicated script was applied as previously reported [31] to evaluate TFEB nuclear translocation.

## 3. Results

### 3.1. TFEB Transcriptionally Regulates p21/WAF1 Expression

**Figure 1:**
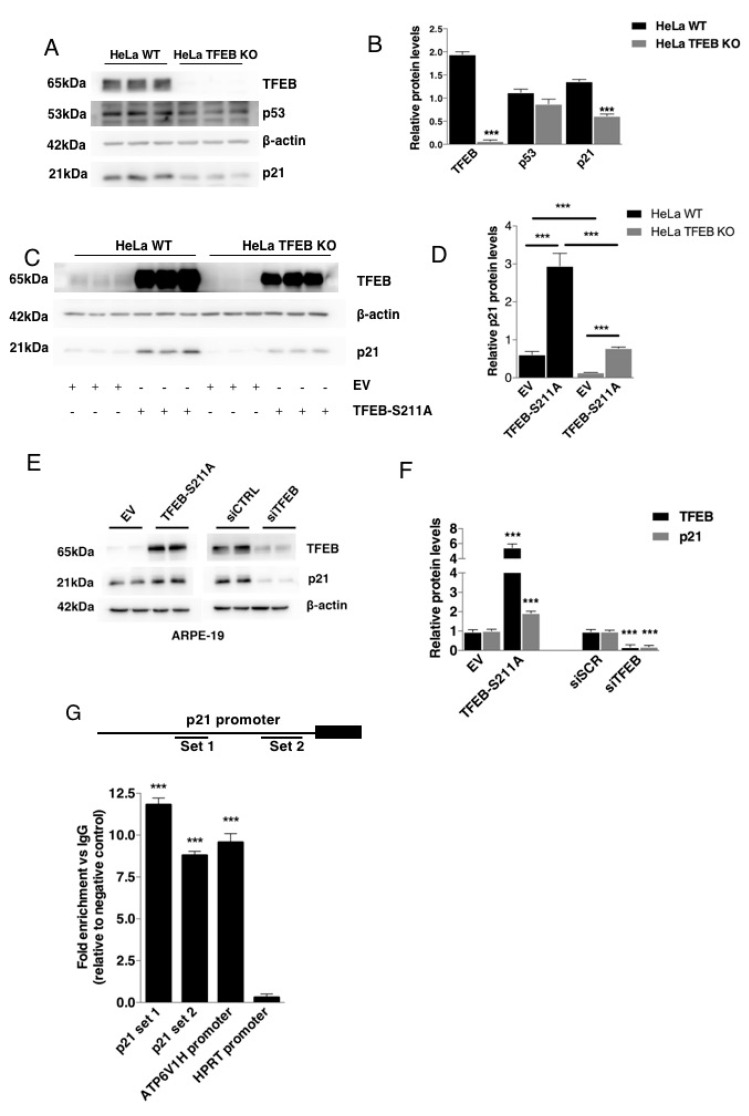
p21 expression is regulated by TFEB. (**A**,**B**) Western blot analysis and quantification of p21, TFEB, and p53 protein levels in HeLa WT cells compared with HeLa TFEB KO cells. β-actin protein levels were used as a loading control. (**C**,**D**) Western blot analysis and quantification of p21 protein levels in HeLa WT compared with TFEB KO cells after the transfection with either an empty vector (3xflagCMV14) or a plasmid encoding constitutive nuclear-localized TFEB (TFEB S211Ax3flag). β-actin protein levels were used as loading control. (**E**,**F**) Western blot analysis and quantification of p21 and TFEB protein levels in Arpe-19 after the transfection with either an empty vector (3xflagCMV14) or a plasmid encoding constitutive nuclear-localized TFEB (TFEB S211Ax3flag) (Left) or transfected with a scrambled sequence siRNA (siSCR) or a siRNA against TFEB (siTFEB) (Right). β-actin protein levels were used as loading control. (**G**) TFEB binding to p21 promoter revealed by chromatin immunoprecipitation (ChIP) assay. Histogram shows fold change in immunoprecipitated chromatin using anti-flag antibody vs IgG in TFEB S211A 3xflag-transfected HeLa cells relative to 3xflagCMV14-transfected HeLa cells (negative control). Chromatin corresponding to the p21 promoter was detected by two different sets of primers. Primers for ATP6V1H and HPRT promoters were used as positive and negative controls, respectively, of chromatin immunoprecipitation along with TFEB. Data are represented as mean ± SEM of three independent experiments (protein). *** *p* < 0.001 (two-tailed Student’s t-test).

Recent evidence suggests that depletion of TFEB/TFE3 transcription factors can dysregulate the expression of genes involved in the DNA-damage response induced by etoposide treatment [26]. Thus, we asked whether the modulation of TFEB levels could influence the expression of cell cycle proteins. To this aim, we compared the basal expression of a set of cell cycle proteins in WT cells or cells depleted of TFEB (HeLa TFEB KO cells) by genome editing [32]. While the levels of most cell cycle proteins did not change significantly, we found a dramatic downregulation of p21 at the level of both mRNA and protein in HeLa TFEB KO cells (Figure 1A–D, Supplementary Figure S1A,B). Conversely, the overexpression of a constitutively active nuclear form of TFEB (TFEB-S211A) increased both mRNA and protein expression of p21 in WT HeLa cells as well as rescued p21 expression on HeLa TFEB KO cells (Figure 1C,D, Supplementary Figure S1C). Similar modulation was observed in a non-cancer cell line, the human retinal pigmented epithelial cell ARPE-19 (Figure 1E,F). Most importantly, by using chromatin immunoprecipitation (ChIP) assay, we found that TFEB binds to the promoter of p21 in HeLa cells transfected with a plasmid encoding TFEB-S211A (Figure 1G). We confirmed the presence of one MiT motif in this region as well as the putative binding motif for well-known factors that binds the promoter of p21 including p53, VDR, and SP1 [33,34,35,36] (Supplementary Figure S1D). Together, these results indicate that TFEB positively regulates the basal expression of the cell cycle inhibitor p21 by direct binding to its promoter.

### 3.2. TFEB-Mediated Induction of p21 Requires p53

**Figure 2:**
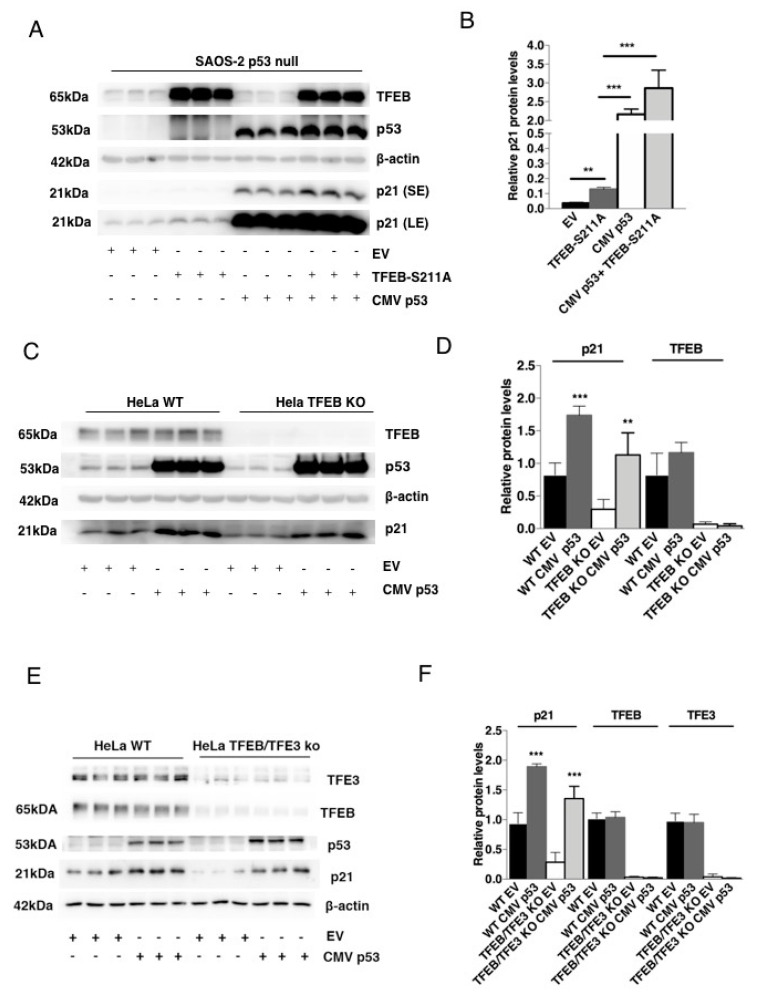
p53 and TFEB regulate p21 expression. (**A**,**B**) Western blot analysis and quantification of p21 protein levels in SAOS-2 p53 null cells after transfection with an empty vector (3xflagCMV14), a plasmid encoding TFEB S211Ax3flag, a p53-encoding plasmid or the combination of both p53- and TFEB S211A-encoding plasmids. β-actin protein levels were used as loading control. SE (Short exposure); LE (Long exposure). (**C**,**D**) Western blot analysis and quantification of TFEB and p21 protein levels in HeLa WT compared with TFEB KO cells after transfection with either an empty vector (3xflagCMV14) or a plasmid encoding p53. β-actin protein levels were used as loading control. (**E**,**F**) Western blot analysis and quantification of TFEB, TFE3, and p21 protein levels in HeLa WT compared with TFEB/TFE3 KO cells after transfection with either an empty vector (3xflagCMV14) or a plasmid encoding p53. β-actin protein levels were used as loading control. Data are represented as mean ± SEM of three independent experiments (protein) or two independent experiments (mRNA). ** *p* < 0.01, *** *p* < 0.001 (two-tailed Student’s t-test).

p21 has been originally identified as a downstream effector of the tumor suppressor transcription factor p53 [5]. p53 activates essential target genes involved in cell cycle arrest, DNA repair, and apoptosis during the DNA-damage response (DDR) [37]. Interestingly, recent work indicates that TFE3 and TFEB can contribute to sustain a p53-dependent response upon genotoxic stress by etoposide [26]. Therefore, we asked whether the modulation of p21 by TFEB requires p53 expression. While in WT cells, TFEB overexpression elevates both the mRNA and protein of p21 without significantly altering p53 protein levels (Supplementary Figure S2A–C), the TFEB-mediated induction of p21 was almost completely inhibited in the p53 null cell line (SAOS-2 p53-null) (Figure 2A,B and Supplementary Figure S2D). As expected, the overexpression of p53 was able to rescue p21 protein and mRNA levels in p53 null cells, and we also observed a further increase of p21 by co-expressing both p53 and TFEB S211A (Figure 2 A,B and Supplementary Figure S2D). Conversely, the overexpression of p53 did not modify TFEB protein levels in HeLa WT cells but increased p21 in both HeLa WT and HeLa TFEB KO cells (Figure 2 C,D, Supplementary Figure S2E). Similarly, p53 overexpression was able to induce p21 protein in HeLa cells double KO for TFEB and TFE3 (Figure 2 D–F). Thus, we can conclude that p53 is required for the induction of TFEB-dependent p21 expression.

### 3.3. p21 Modulation in Response to DNA Damage Requires TFEB

**Figure 3:**
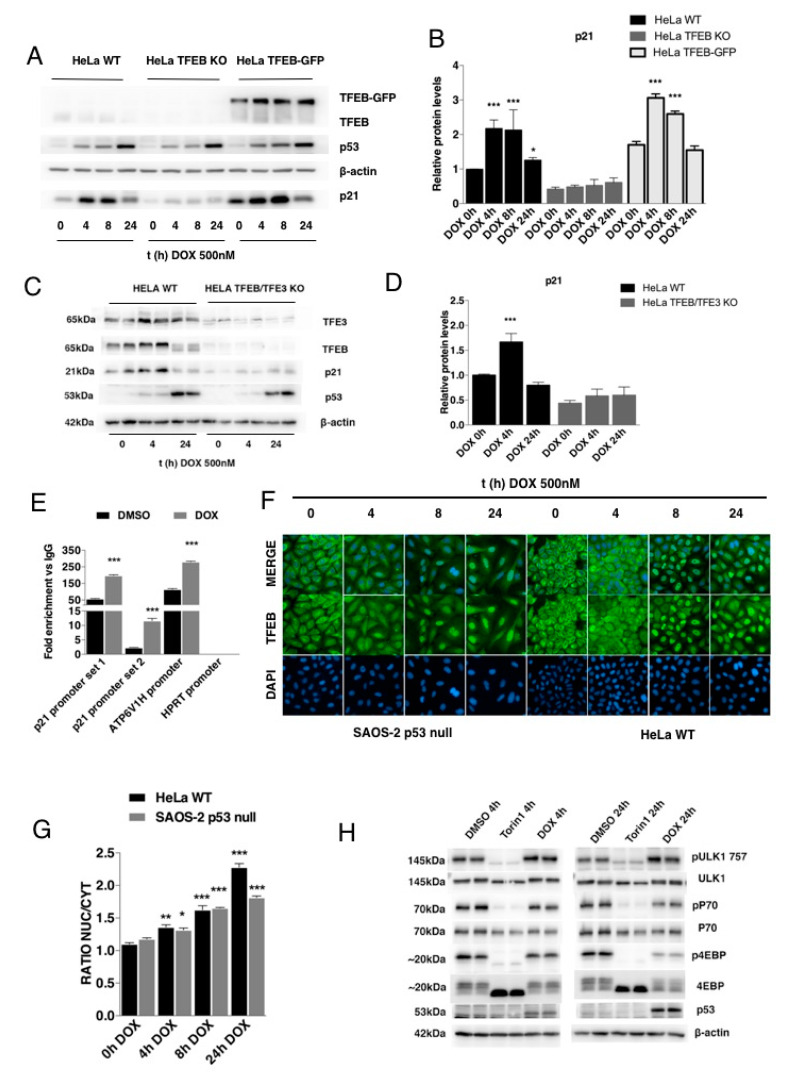
p21 upregulation in response to DNA damage requires TFEB activation. (**A**) Western blot analysis of the protein content of p21, p53, and TFEB in HeLa WT, TFEB KO, and TFEB-GFP after treatment with doxorubicin (DOX) at 0.5 µM for 0–24 h. β-actin protein levels were used as loading control. (**B**) Quantification of p21 protein levels in HeLa WT, TFEB KO, and TFEB-GFP after treatment with doxorubicin (DOX) at 0.5 µM for 0–24 h. (**C**) Western blot analysis of the protein content of p21, p53, TFE3, and TFEB in HeLa WT, TFEB/TFE3 KO after treatment with doxorubicin (DOX) at 0.5 µM for 0–24 h. β-actin protein levels were used as a loading control. (**D**) Quantification of p21 protein levels in HeLa WT, TFEB/TFE3 KO after a treatment with doxorubicin (DOX) at 0.5 µM for 0–24 h. (**E**) Analysis of TFEB binding to p21 promoter by chromatin immunoprecipitation (ChIP) assay in HeLa WT cells stably expressing TFEB 3xflag upon treatment with either DMSO or doxorubicin (DOX) at 0.5 µM for 4 h. Histogram shows fold change in immunoprecipitated chromatin using anti-flag antibody vs IgG. Chromatin corresponding to the p21 promoter was detected by two different sets of primers. Primers for ATP6V1H and HPRT promoters were used as positive and negative controls, respectively, of chromatin immunoprecipitation along with TFEB. (**F,G**) TFEB nuclear translocation measurement after the treatment with doxorubicin (DOX) at 0.5 µM for 0–24 h in HeLa WT and SAOS-2 p53-null cells. Images were acquired by using an Opera Automated Confocal Microscopy and subsequently analyzed through the Columbus Image data storage and analysis system. (**F**) Western blot analysis of the protein content of pULK757/ULK, pP70/P70, p4EPB/4EPB, p21, and p53 in HeLa WT after treatment with doxorubicin (DOX) at 0.5 µM for 4–24 h and compared with a treatment of Torin1 0.3 µM as positive control of mTOR inhibition. β-actin protein levels were used as loading control. Data are represented as mean ± SEM of three independent experiments. * *p* < 0.05, ** *p* < 0.01, *** *p* < 0.001 vs. time 0 h (two-tailed Student’s t-test).

Intrigued by the TFEB-mediated modulation of p21, we tested whether genotoxic induction using the chemotherapeutic agent doxorubicin could activate the TFEB-p21 pathway. Doxorubicin causes severe DNA double-strand breaks, promoting p53-dependent induction of p21 and leading to a block of the cell in the G2-phase of the cell cycle [38]. As expected, the treatment with doxorubicin caused a time-dependent increase of p53 and p21 expression that reaches the maximal induction at 8 hours to then decay at 24 h, most likely via the proposed degradation by the proteasome [39] (Figure 3A). Interestingly, while doxorubicin-mediated upregulation of p53 was very similar in both WT and HeLa TFEB KO cells, the induction of p21 was severely impaired in TFEB KO cells (Figure 3A,B). Similar results were obtained using HeLa cells double KO for TFEB and TFE3 (Figure 3C,D), suggesting that the doxorubicin-mediated upregulation of p21 requires TFEB. Conversely, HeLa cells stably overexpressing TFEB-GFP protein showed a better response, elevating p21, upon doxorubicin treatment (Figure 3A). By using ChIP analysis, we also confirmed an increase of TFEB binding to the p21 promoter (Figure 3E) and p21 mRNA elevation upon doxorubicin treatment (Supplementary Figure S3A), suggesting a transcriptional control.

Then, we investigated whether doxorubicin could activate TFEB by looking at the subcellular localization of endogenous TFEB [31]. We found a progressive induction of TFEB nuclear localization during the treatment with doxorubicin (Figure 3F,G). Recent evidence suggests that the DNA damage agent etoposide induces TFEB nuclear translocation through the inhibition of mTORC1 in a p53 dependent manner [26]. However, we found that at two different time-points (4 and 24 h, respectively), the treatment with doxorubicin was not able to inhibit significantly mTORC1 by looking at the phosphorylation of various of its substrates (Figure 3H). In addition, doxorubicin treatment was able to induce endogenous TFEB nuclear translocation in SAOS-2 p53-null cells (Figure 3F,G). As expected, instead, doxorubicin was not inducing p21 in these cells (Supplementary Figure S3B). Collectively, these results strongly indicate that doxorubicin induces TFEB nuclear translocation through a mechanism that is independent of mTOR and p53.

### 3.4. Lack of TFEB Promotes a Faster Arrest in G2/M Phase in Response to DNA Damage

**Figure 4:**
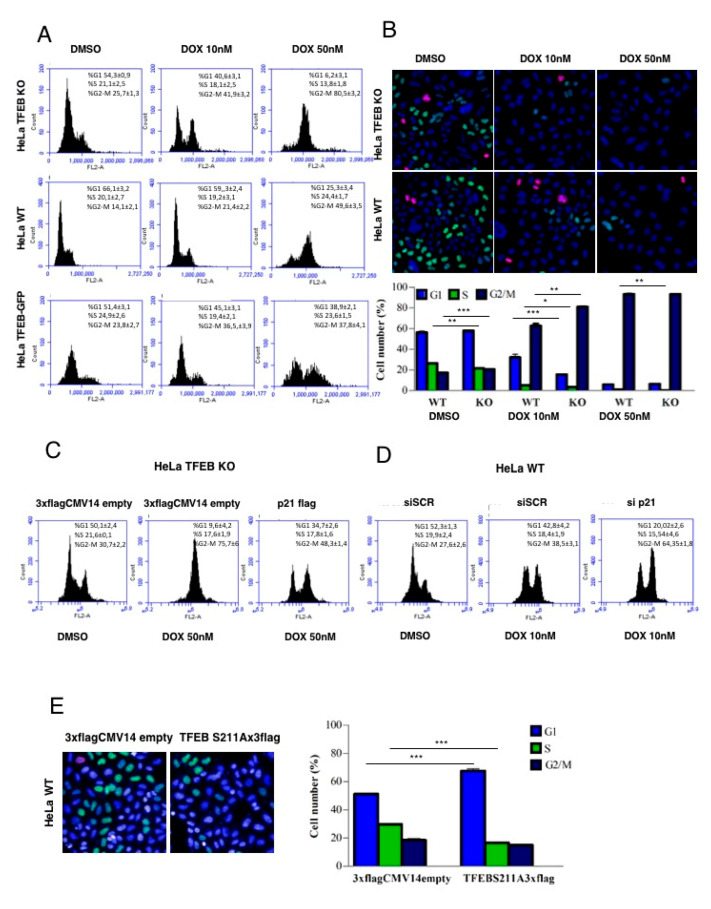
TFEB depletion leads to a faster arrest in the G2 phase in response to DNA damage. (**A**) Representative histograms showing flow cytometry analysis of cell cycle in HeLa WT compared with HeLa TFEB KO and HeLa TFEB-GFP after the treatment with doxorubicin (10 nM and 50 nM) for 24 h. (**B**) High Content Imaging analysis of the percentage of cells in G1, S, and G2/M phases in HeLa WT compared with HeLa TFEB KO after the treatment with doxorubicin (10 nM and 50 nM) for 24 h. (**C**) Representative histograms showing flow cytometry analysis of cell cycle in HeLa TFEB KO overexpressing p21 compared with empty vector-transfected cells after the treatment with doxorubicin (50 nM) for 24 h. (**D**) Representative histograms showing flow cytometry analysis of cell cycle in HeLa WT cells transfected with either non-targeting siRNA (siSCR) or p21-targeting siRNA (sip21) for 72 h, and treated with doxorubicin (10 nM) the last 24 h. (**E**) High Content Imaging analysis of the percentage of cells in G1, S, and G2/M phases in HeLa WT cells transfected with an empty vector or a plasmid encoding TFEB S211A for 24 h. Data are represented as mean ± SEM of three independent experiments * *p* < 0.05, ** *p* < 0.01, *** *p* < 0.001 (two-tailed Student’s t-test).

Then, we asked whether the modulation of TFEB protein levels could be relevant modulating the cell cycle of cancer cells in untreated conditions or after the treatment with doxorubicin, which causes a block of G2-phase of the cell cycle [38] (Figure 4A). We found that cells depleted of TFEB, and therefore having lower basal levels of p21 (Figure 1A,B), undergo a faster G2-phase arrest after the treatment with two different concentrations of doxorubicin for 24 h (Figure 4A). Similar results were obtained at 48 h (Supplementary Figure S4). Conversely, in HeLa cells stably overexpressing TFEB, and therefore having higher levels of p21 (Figure 1C,D), we observed a slower G2-phase arrest upon doxorubicin treatment (Figure 4A). These results indicated that TFEB might be involved in the progression of the cell cycle through the modulation of p21 levels. This idea is supported by the significant reduction in the percentage of HeLa cells in the S-phase with a significant increase in the G2/M-phase when TFEB is not expressed, as revealed the analysis of the phases of the cell cycle by High Content Imaging (Figure 4B). In line with this finding, the over-expression of p21 in TFEB KO cells leads to a delay in the progression of the cell cycle to the G2-phase after doxorubicin treatment (Figure 4C). Conversely, in HeLa WT cells, reduction of p21 levels by specific siRNAs leads to a faster arrest in the G2-phase, mimicking the effect of doxorubicin in TFEB KO HeLa cells (Figure 4D). Moreover, overexpression of the TFEB S211A mutant, which considerably elevates p21 levels, promoted a significant increase in the percentage of cells in the G1-phase (Figure 4E). Together, these results indicate that TFEB plays a major role in doxorubicin-mediated DDR by modulating the cell cycle through the expression of p21 protein.

### 3.5. TFEB Promotes Cancer Cell Survival Upon DNA Damage Through p21 Upregulation

**Figure 5:**
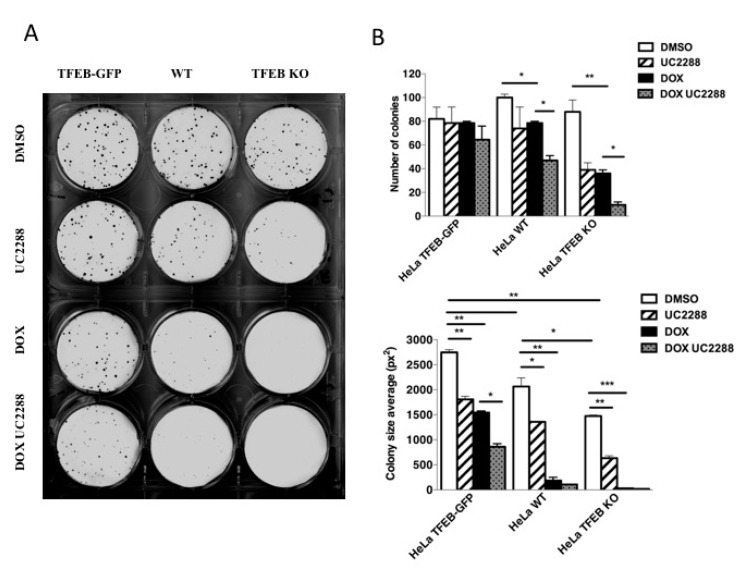
TFEB promotes cancer cell survival upon DNA damage through p21 upregulation. (**A**) Representative pictures of colony formation capacity in HeLa TFEB-GFP, WT, and TFEB KO cells in normal conditions (control DMSO) or upon the treatment with doxorubicin 10 nM for 8 days, alone or in combination with UC2288 2.5 µM. (**B**) Analysis of colony number (top) and size (bottom) in upon different treatments. Quantification was performed by Image J, taken into consideration colonies bigger than 4px^2^ and with a roundness higher than 0.3. Data are represented as mean ± SEM of two independent experiments * *p* < 0.05, ** *p* < 0.01, *** *p* < 0.001 (two-tailed Student’s t-test).

To evaluate the role of the TFEB-p21 pathway in cancer cell survival, we performed colony formation assays using cells with different levels of TFEB and p21 (WT, TFEB KO, and HeLa cells stably overexpressing TFEB-GFP), in presence or absence of doxorubicin. While the number of colonies formed was similar in all cell lines treated with DMSO, the average size of these colonies was dramatically reduced in cells depleted of TFEB (Figure 5A,B). More interestingly, treatment with doxorubicin reduced both number and size of colonies in a higher extent in cells with lower levels of TFEB-p21 expression. These results suggest that higher levels of TFEB expression positively correlate with better cell survival in the presence of genotoxic stress. To check whether these effects are mediated by p21 upregulation, we treated cells with the p21 inhibitor UC2288, which induces a decrease in both p21 mRNA and protein levels [40] (Supplementary Figure S3A,B). We found that the treatment with UC2288 alone for 8 days significantly reduces the number of colonies in HeLa TFEB KO cells and the average size of these colonies in all cell lines treated. Interestingly, the co-treatment with doxorubicin and UC2288 significantly reduces the number of colonies in HeLa WT and Hela TFEB KO but not in cells overexpressing TFEB (Figure 5A,B). In addition, the average size of the colonies was progressively reduced in all cell lines, although stably HeLa cells overexpressing TFEB were less sensitive to the co-treatment (Figure 5A,B). We further study the role of the TFEB-p21 pathway in cancer cell survival by testing the efficacy of doxorubicin to promote caspase-3 cleavage at two different concentrations (Supplementary Figure S5C,D). We found that doxorubicin was less effective cleaving caspase-3 in cells overexpressing TFEB as compared with WT and TFEB KO cells, and only the highest concentration of doxorubicin weakly induces caspase-3 cleavage in HeLa TFEB-GFP cells (Supplementary Figure S5C,D). Altogether, these results unveil the role of TFEB as a pro-survival factor in cancer cells through the upregulation of p21.

## 4. Discussion

In normal cells, the induction of DNA damage or other stress signals activate the tumor suppressor p53, leading to transient expression of the cyclin-dependent kinase inhibitor (CKI) p21. In these conditions, p21 plays a role as an antiproliferative effector and mediator of cellular senescence. However, recent evidence suggests that p21 can acquire either tumor suppressor or oncogenic properties depending on cell type, cellular localization, p53 status, and the type and level of genotoxic stress. In cancer cells, the overexpression of p21 may promote the generation of cancer cell clones that escape from senescence, harbor extreme genome instability, and present a drug-resistant phenotype [7]. Thus, a novel therapeutic strategy may consist of targeting specific cancer cell types in which p21 is upregulated. Here, we found that p21 is a transcriptional target of TFEB, a master regulator of lysosomal function [28,30]. We found that TFEB depletion dramatically downregulates the basal expression of p21 protein in both normal and cancer cell types. Conversely, the overexpression of TFEB elevates both mRNA and protein levels of p21. Interestingly, a tissue-restricted member of the TFEB family, MITF, is a well-known activator of p21 expression, a process that may contribute to the cell cycle exit and activation of the differentiation program of melanocyte lineage [1]. Importantly, MITF can act as an amplified oncogene in a fraction of human melanomas and that it also has an oncogenic role in human clear cell sarcoma. Similarly, aberrant overexpression of TFEB has been reported in patients with renal cell carcinoma [41]. Whether or not the modulation of p21 also plays a role in the oncogenic activity of TFEB/TFE3-induced renal carcinoma, will be the subject of future studies.

Then, we found that one of the major regulators of p21, the tumor suppressor p53 [16,27,28,42,43,44], is required for TFEB-mediated elevation of p21. Interestingly, a residual but significant activation of p21 was observed upon the overexpression of TFEB in p53 depleted background. This observation may indicate a p53-independent mechanism of p21 induction through TFEB that needs further investigation. Although not at the same level that its overexpression in WT cells, exogenous p53 can increase p21 protein levels in TFEB KO as well as in double TFEB/TFE3 KO cells, indicating that p53 can still induce p21 in a TFEB-independent manner. In addition, co-expression of p53 and TFEB resulted in a synergic effect elevating p21. Thus, even though p53 may not require TFEB to induce p21 expression, it may cooperate with TFEB to modulate p21 levels in some cancer types or under specific stimuli.

Several genotoxic stressors inducing DDR such as etoposide, cisplatin, UVC, and doxorubicin can induce TFEB nuclear translocation [26,45]. Indeed, we found that TFEB activation is essential to induce p21 up-regulation upon treatment with the DNA-damage agent doxorubicin. Cell cycle analysis of cells expressing different levels of TFEB protein upon doxorubicin treatment revealed that transition to G2 arrest was faster in TFEB depleted cells, where p21 levels are lower, and that p21 overexpression can rescue this phenotype. In addition, siRNA-mediated p21 reduction in HeLa WT cells accelerated G2 arrest. As expected, when p21 transcription was notably activated, through TFEB S211A overexpression, we observed a block in the G1 phase. Interestingly, doxorubicin was able to induce TFEB nuclear translocation independently of p53 expression and mTORC1 activity, while different genotoxic stressors, such as etoposide, induce TFEB/TFE3 activation at least in part through p53-mediated mTORC1 inhibition [26]. Thus, while our findings suggest that TFEB can regulate the expression of p21 in a p53-dependent manner, the mechanism of action of different genotoxic drugs inducing DDR may vary activating TFEB through different pathways.

Given the implications of p21 in chemotherapeutic drug resistance [42,43], we evaluated the contribution of TFEB-mediated p21 upregulation in the capacity of cancer cells to survive upon the treatment doxorubicin. Surprisingly, TFEB expression was correlated with higher survival capacity not only upon doxorubicin treatment, but also in normal conditions. Moreover, this effect was mediated, at least in part, through p21 upregulation, as demonstrated by the reduction in the formation and the average size of the colonies upon treatment with the p21-selective inhibitor UC2288. Interestingly, also MITF has been involved in the resistance to UVC-induced cell death through p21 upregulation [2]. Altogether and consistently with our findings, the attenuation of p21 expression combined with DNA-damaging drugs could be useful in cancer therapy. Collectively, the uncovered TFEB-p21 pathway may represent a new target for therapeutic intervention in different kinds of cancers.

## Supplementary Materials

The following are available online at https://www.mdpi.com/2073-4409/9/5/1186/s1, Figure S1: (A) Western blot analysis of pWee (Ser642), Myt1, Cyclin B1, AuroraB, p27, pHH3 (Ser10), Cyclin E2, pCdc2 (Tyr15), Cyclin A2, and p18 protein levels in HeLa WT cells compared with HeLa TFEB KO. β-actin protein levels were used as a loading control. (B) Quantification of p21 mRNA in HeLa WT compared with TFEB KO cells. (C) Quantification of p21 and TFEB mRNA in HeLa TFEB KO cells after the transfection with either an empty vector (3xflagCMV14) or a plasmid encoding constitutive nuclear-localized-TFEB (TFEB S211Ax3flag). Data are represented as mean ± SEM of three independent experiments. ** p < 0.01, *** p < 0.001 (two-tailed Student’s t-test). (D) Schematic representation of the region of p21 promoter used in this study. Figure S2: (A,B) Western blot analysis and quantification of p21 and p53 in HeLa WT after the transfection with either an empty vector (3xflagCMV14) or a plasmid encoding constitutive nuclear-localized-TFEB (TFEB S211Ax3flag). β-actin protein levels were used as a loading control. (C) p21 mRNA levels in HeLa WT after the transfection with either an empty vector (3xflagCMV14) or a plasmid encoding constitutive nuclear-localized-TFEB (TFEB S211Ax3flag). (D) Quantification of p21 and TFEB mRNA in SAOS-2 p53 null cells after transfection with an empty vector (3xflagCMV14), a plasmid encoding TFEB S211Ax3flag, a p53-encoding plasmid, or the combination of both p53- and TFEB S211A-encoding plasmids. (E) Quantification of p21 and TFEB mRNA in HeLa WT compared with TFEB KO cells after transfection with either an empty vector (3xflagCMV14) or a plasmid encoding p53. Data are represented as mean ± SEM of three independent experiments. ** p < 0.01, *** p < 0.001 vs. DMSO (two-tailed Student’s t-test). Figure S3: (A) p21 mRNA levels in HeLa WT cells stably expressing TFEB 3xflag upon treatment with either DMSO or doxorubicin (DOX) at 0.5 µM for 4 h. (B) Western blot analysis of p21 in SAOS-2 p53 null cells upon treatment with either DMSO or doxorubicin (DOX) at 0.5 µM for 4 h, HeLa WT were used as doxorubicin control. Data are represented as mean ± SEM of three independent experiments. *** p < 0.001 (two-tailed Student’s t-test). Figure S4: Representative histograms showing flow cytometry analysis of cell cycle in HeLa WT compared with HeLa TFEB KO after the treatment with doxorubicin (10 nM and 50 nM) for 48 h.
