# Supplementary material for: TFEB Modulates p21/WAF1/CIP1 during the DNA Damage Response
Source: Cells. 2020 May 10;9(5):1186. doi: 10.3390/cells9051186 (PMC7290768; doi:10.3390/cells9051186)
Supplement: Supplementary file 1 [file cells-09-01186-s001.pdf]

TFEB modulates p21/WAF1/CIP1 during the DNA damage response

Sandra Pisonero-Vaquero[1#], Chiara Soldati[1#], Marcella Cesana[1,2], Andrea Ballabio[1,3,4,5], Diego Luis Medina[1,3*]

[1] Telethon Institute of Genetics and Medicine (TIGEM), High Content Screening facility, Via Campi Flegrei 34, 80078 Pozzuoli (NA), Italy; spisv@unileon.es; c.soldati@tigem.it; m.cesana@tigem.it; ballabio@tigem.it; medina@tigem.it)

[2] Deparment of Advance Biomedical Science, Federico II University, Naples, Italy

[3] Medical Genetics Unit, Department of Medical and Translational Science, Federico II University, Naples, Italy

[4] Baylor College of Medicine, Houston, Texas, USA;

[5] Jan and Dan Duncan Neurological Research Institute, Texas Children's Hospital, Houston, Texas, USA;

* Correspondence: medina@tigem.it

# equal contribution

Received: 7 April 2020; Accepted: date; Published: date

Supplementary Materials:

Supplementary figure 1

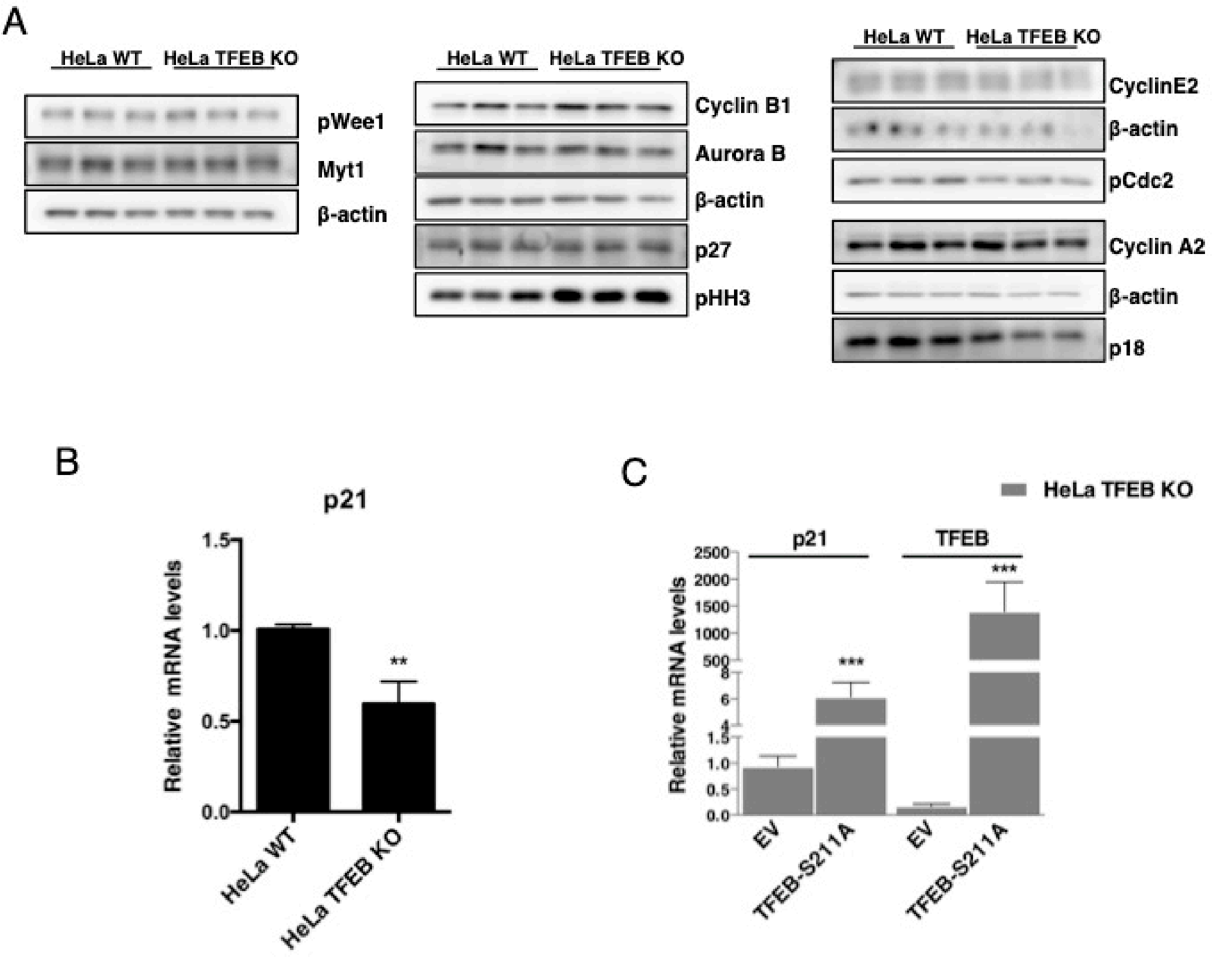

D

chr6:36677814-36678816:TTCCCCAGCAGTGTATACGGGCTATGTGGGGAGTATTCAGGAGACAG**ACAACTCACTCGTCAAATCC**
TCCCCTTCCTGGCCAACAAAGCTGCTGCAACCACAGGGATTTCTTCTGTTCAGGTGAGTGTAGGGTGTAGGGAGATTGGTTCAAT
GTCCAATTCTTCTGTTTCCCTGGAGATCAGGTTGCCCTTTTTTGGTAGTCTCTCCAATTCCCTCCTTCCCGGAAGCATGTGACAATCAA
CAACTTTGTATACTTAAGTTCAGTGGACCTCAATTTCCT..//...GCGCACCAACGCAGGCGAGGGACTGGGGGAGGAGGGAAGTGCC
CTCCTGCAGCACGCGAGGTTC**CGGGACCGGCTGGCCTG**CTGGAACTCGGCCAGGCTCAGCTGGCTCGGCGCTGGGCAGCCAGGAG
CCTGGGCCCCGGGGAGGGCGGTCCCGGGCGGCGCGGTGGGCCGAGCGCGGGTCCCGCCTCCTTG**AGGCGGGCCCGGGCGGGGC**
GGTTGTATATCAGGGCCGCGCTGAGCTGCGCCAGCTGAGGTGTGAGCAGCTGCCGAAGTCAGTTCCTTGTGGAGCCGGAGCTGG
GCGCGGATTCGCCGAGGCACCGAGGCACTCAGAGGAGGTGAGAGAGCGGCGGCAG

IN RED: sequences amplified by set 1 and set 2 (ChiP)
AGCATGTGAC : MiT °
AGGCGGGCCCGGGCGGGGCG : P53 °°
CGGGACCGGCTGGCCTG : VDR °°°
cccgGGCGGggcg : SP1 °°°°
(°Hemesath et al, 1994; °° Laptenko et al, 2011; °°°Saramäki et al, 2006; °°°°Suske et al, 1999)

Supplementary Figure S1. (**A**) Western blot analysis of pWee (Ser642), Myt1, Cyclin B1, AuroraB, p27, pHH3 (Ser10), Cyclin E2, pCdc2 (Tyr15), Cyclin A2, and p18 protein levels in HeLa WT cells compared with HeLa TFEB KO. β-actin protein levels were used as a loading control. (**B**) Quantification of p21 mRNA in HeLa WT compared with TFEB KO cells. (**C**) Quantification of p21 and TFEB mRNA in HeLa TFEB KO cells after the transfection with either an empty vector (3xflagCMV14) or a plasmid encoding constitutive nuclear-localized-TFEB (TFEB S211Ax3flag). Data are represented as mean ± SEM of three independent experiments. ***p* < 0.01, ****p* < 0.001 (two-tailed Student's t-test). (**D**) Schematic representation of the region of p21 promoter used in this study.

Supplementary Figure 2

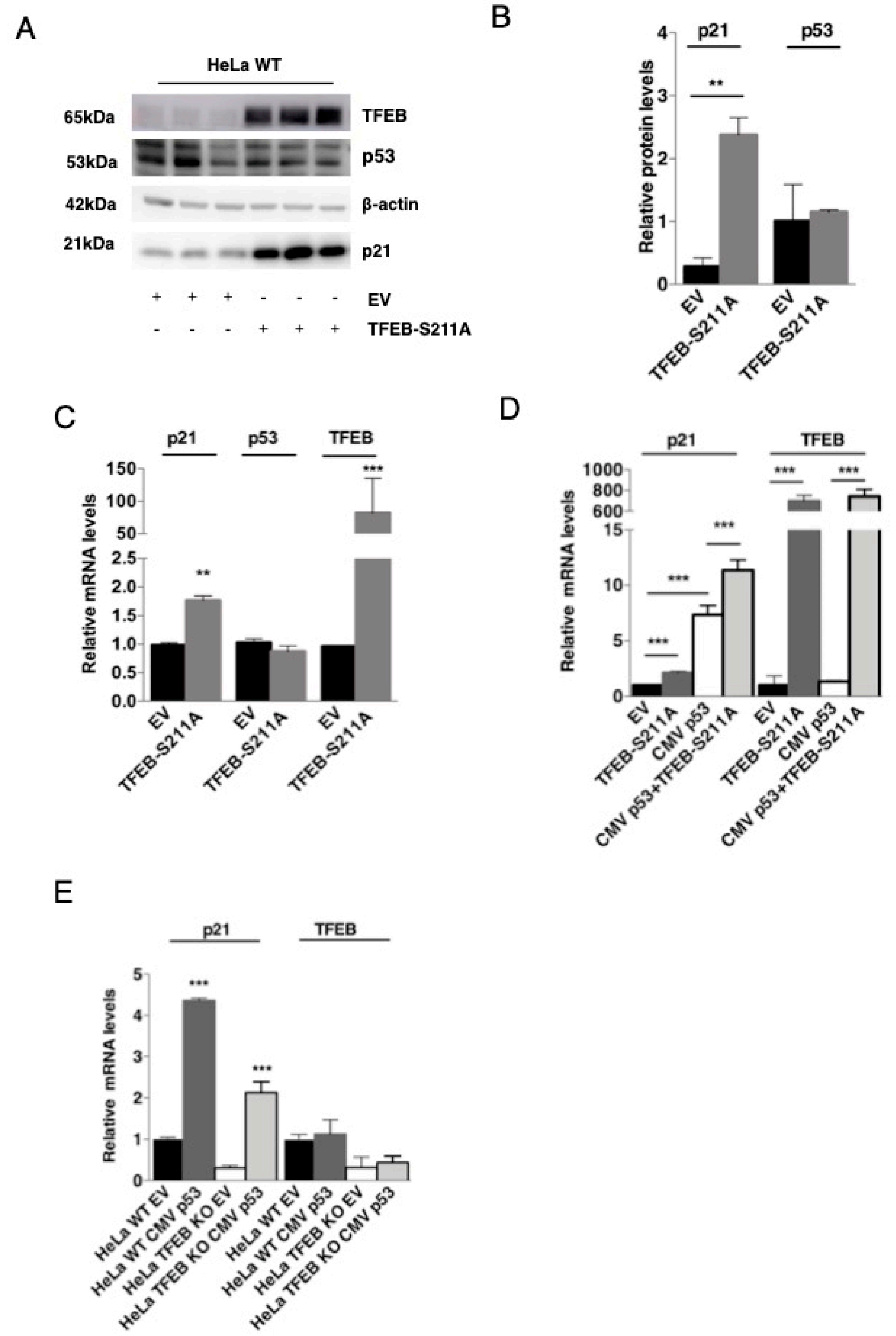

Supplementary Figure S2. (**A**,**B**) Western blot analysis and quantification of p21 and p53 in HeLa WT after the transfection with either an empty vector (3xflagCMV14) or a plasmid encoding constitutive nuclear-localized-TFEB (TFEB S211Ax3flag). β-actin protein levels were used as a loading control. (**C**) p21 mRNA levels in HeLa WT after the transfection with either an empty vector (3xflagCMV14) or a plasmid encoding constitutive nuclear-localized-TFEB (TFEB S211Ax3flag). (**D**) Quantification of p21 and TFEB mRNA in SAOS-2 p53 null cells after transfection with an empty vector (3xflagCMV14), a plasmid encoding TFEB S211Ax3flag, a p53-encoding plasmid, or the combination of both p53- and TFEB S211A-encoding plasmids. (**E**) Quantification of p21 and TFEB mRNA in HeLa WT compared with TFEB KO cells after transfection with either an empty vector (3xflagCMV14) or a plasmid encoding p53. Data are represented as mean ± SEM of three independent experiments. $^{**}p < 0.01$, $^{***}p < 0.001$ vs. DMSO (two-tailed Student's t-test).

Supplementary Figure 3

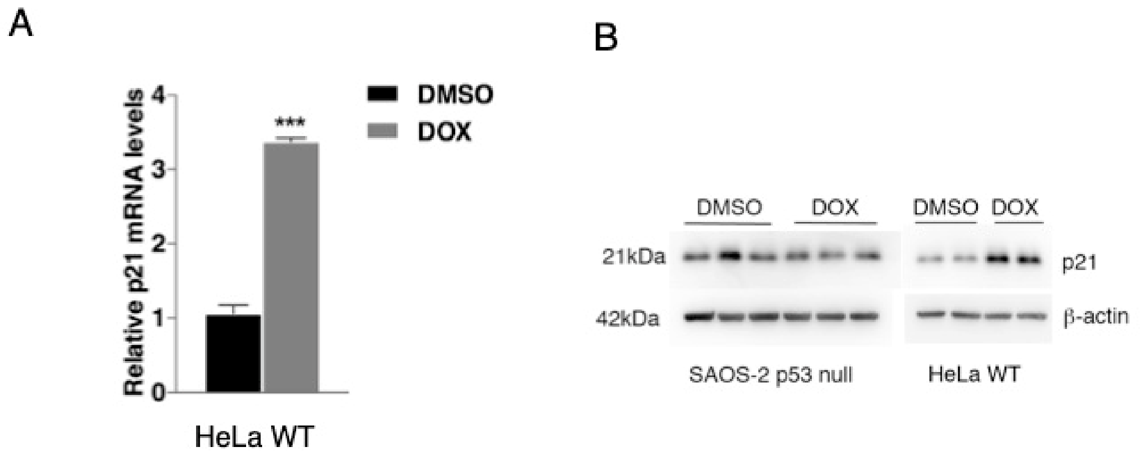

Supplementary Figure S3. (**A**) p21 mRNA levels in HeLa WT cells stably expressing TFEB 3xflag upon treatment with either DMSO or doxorubicin (DOX) at 0.5 µM for 4 h. (**B**) Western blot analysis of p21 in SAOS-2 p53 null cells upon treatment with either DMSO or doxorubicin (DOX) at 0.5 µM for 4 h, HeLa WT were used as doxorubicin control. Data are represented as mean ± SEM of three independent experiments. ****p* < 0.001 (two-tailed Student's t-test).

Supplementary Figure 4

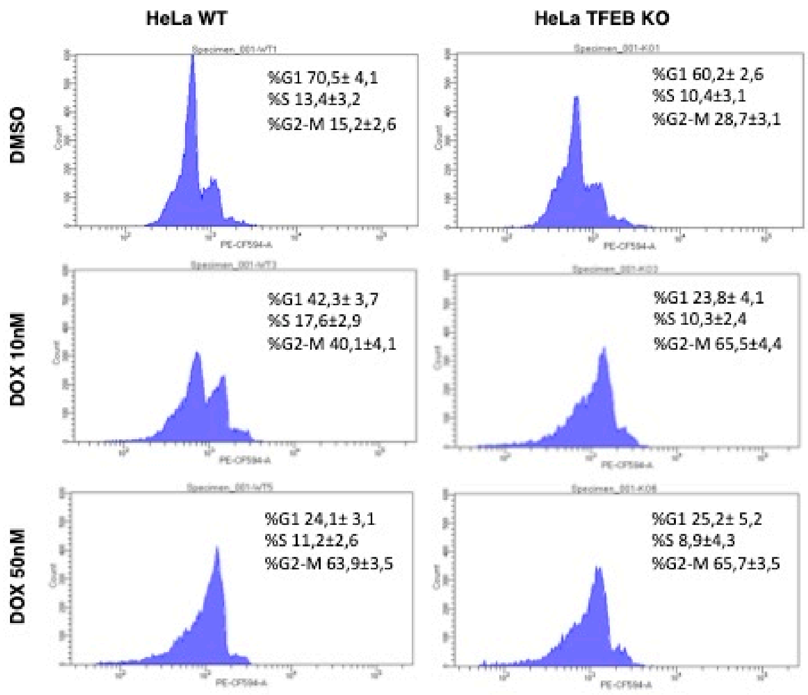

Supplementary Figure S4. Representative histograms showing flow cytometry analysis of cell cycle in HeLa WT compared with HeLa TFEB KO after the treatment with doxorubicin (10 nM and 50 nM) for 48 h.

Supplementary Figure 5

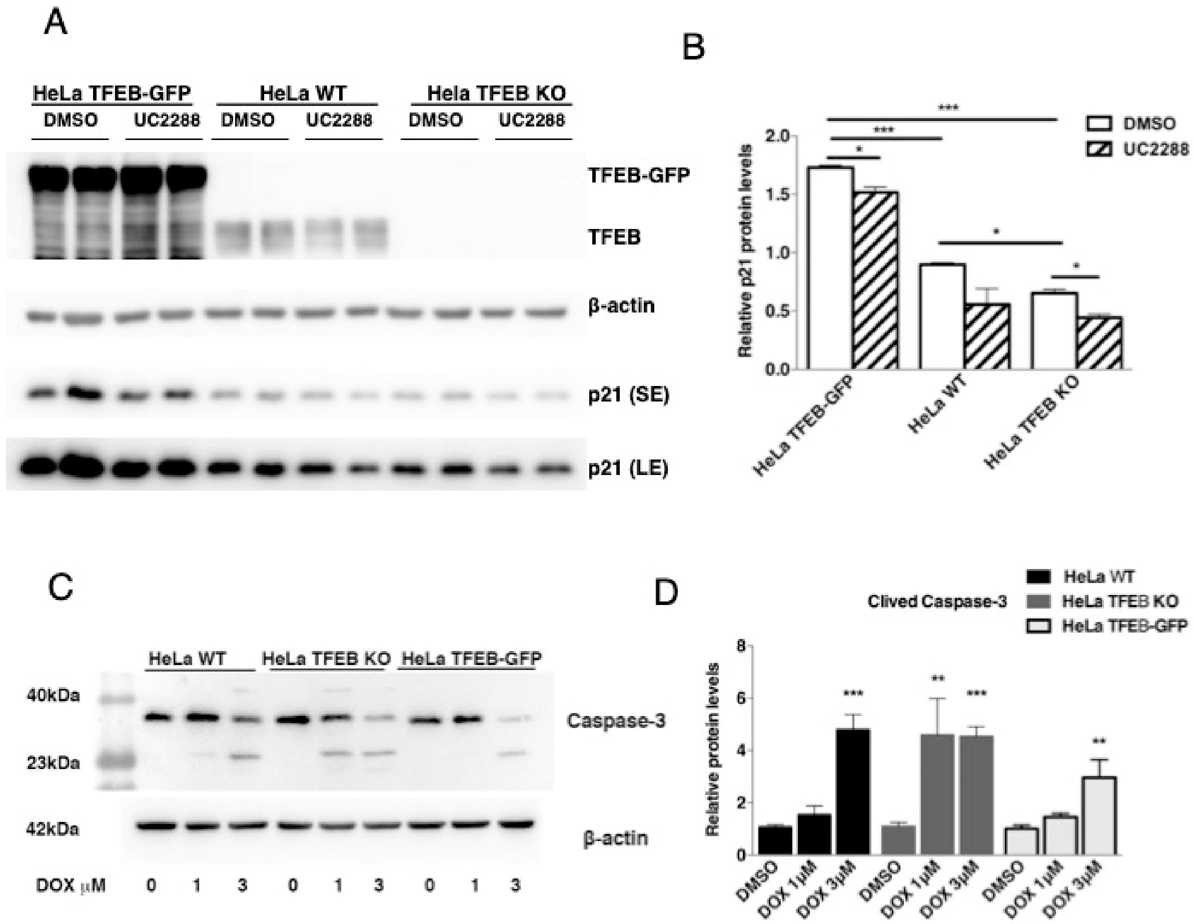

Supplementary Figure 5. Effect of UC2288 on p21 protein levels in HeLa cells expressing different levels of TFEB. (**A**,**B**) Analysis and quantification of p21 protein levels by Western blot in HeLa WT compared with TFEB-GFP overexpressing and TFEB KO cells after treatment for three days with either the p21 inhibitor UC2288 2.5 μM or DMSO 0.05%. β-actin protein levels were used as a loading control. SE (Short exposure); LE (Long exposure). (**C**) Western blot analysis of Caspase-3 and (**D**) quantification of cleaved Caspase-3 in HeLa WT compared with HeLa TFEB KO and HeLa TFEB-GFP cells upon treatment with either DMSO or doxorubicin (DOX) at 1 or 3 μM for 16 h. Data are represented as mean ± SEM of three independent experiments. *$p < 0.05$, **$p < 0.01$ ***$p < 0.001$ (two-tailed Student's t-test).
